# Physical Exercise and Mechanism Related to Alzheimer’s Disease: Is Gut–Brain Axis Involved?

**DOI:** 10.3390/brainsci14100974

**Published:** 2024-09-27

**Authors:** Javier Sanchez-Martinez, Patricio Solis-Urra, Jorge Olivares-Arancibia, Julio Plaza-Diaz

**Affiliations:** 1 Department of Physical Education and Sports, Faculty of Sport Sciences, Sport and Health University Research Institute (iMUDS), University of Granada, 18071 Granada, Spain; sanchez.javier.andre@gmail.com; 2 Faculty of Education and Social Sciences, Universidad Andres Bello, Viña del Mar 8370134, Chile; 3 AFySE Group, Research in Physical Activity and School Health, School of Physical Education, Faculty of Education, Universidad de Las Américas, Santiago 7500975, Chile; jorge.olivares.ar@gmail.com; 4 Department of Biochemistry and Molecular Biology II, School of Pharmacy, Campus de Cartuja s/n, University of Granada, 18071 Granada, Spain; 5 Instituto de Investigación Biosanitaria IBS.GRANADA, Complejo Hospitalario Universitario de Granada, 18014 Granada, Spain; 6 Children’s Hospital of Eastern Ontario Research Institute, Ottawa, ON K1H 8L1, Canada

**Keywords:** Alzheimer’s disease, neurodegenerative disorders, physical exercise, gut–brain axis, cognitive functions, brain-derived neurotrophic factor

## Abstract

Background: Alzheimer’s disease is a progressive neurodegenerative disease characterized by structural changes in the brain, including hippocampal atrophy, cortical thinning, amyloid plaques, and tau tangles. Due to the aging of the global population, the burden of Alzheimer’s disease is expected to increase, making the exploration of non-pharmacological interventions, such as physical exercise, an urgent priority. Results: There is emerging evidence that regular physical exercise may mitigate the structural and functional declines associated with Alzheimer’s disease. The underlying mechanisms, however, remain poorly understood. Gut–brain axis research is a promising area for further investigation. This system involves bidirectional communication between the gut microbiome and the brain. According to recent studies, the gut microbiome may influence brain health through modulating neuroinflammation, producing neuroactive compounds, and altering metabolic processes. Exercise has been shown to alter the composition of the gut microbiome, potentially impacting brain structure and function. In this review, we aim to synthesize current research on the relationship between physical exercise, structural brain changes in Alzheimer’s disease, and the gut–brain axis. Conclusions: In this study, we will investigate whether changes in the gut microbiome induced by physical exercise can mediate its neuroprotective effects, offering new insights into the prevention and treatment of Alzheimer’s disease. By integrating findings from neuroimaging studies, clinical trials, and microbiome research, this review will highlight potential mechanisms. It will also identify key gaps in the literature. This will pave the way for future research directions.

## 1. Structural Correlates of Alzheimer’s Disease

Alzheimer’s disease is a progressive neurodegenerative disorder that is the most common cause of dementia in older adults, affecting millions of people worldwide [1,2]. Characterized by the gradual decline in cognitive abilities, memory loss, and behavioral changes, Alzheimer’s disease profoundly impacts both patients and their families [1,2]. The disease progresses from mild cognitive impairment to severe dementia, eventually leading to the loss of basic bodily functions. Various hypotheses have been proposed to explain the onset and progression of Alzheimer’s disease. These include amyloid, cholinergic, inflammatory, tau protein, metal ions, oxidative stress, glutamate excitotoxicity, abnormal autophagy, and the microbiota–gut–brain axis [3]. Among these, the amyloid hypothesis has been particularly influential, suggesting that the accumulation of amyloid-beta (Aβ) peptides in the brain plays a central role in triggering the pathological changes associated with Alzheimer’s disease [1,4].

The brain undergoes profound structural changes driven by several mechanisms, such as neuron cell death, dendritic degeneration, metabolic slowing, and microglial activation, among others [5,6]. These mechanisms are closely related to the disorder’s progressive cognitive decline. One of the most notable changes is the accumulation of Aβ plaques, which are the extracellular deposits of misfolded Aβ peptides [7]. These plaques primarily form in the hippocampus and cortex (regions critical for memory and cognitive function) [8], disrupting neural communication and contributing to synaptic loss. Alongside amyloid plaques, neurofibrillary tangles made of hyperphosphorylated tau protein develop inside neurons [7]. These tangles disrupt the neuronal cytoskeleton, impairing intracellular transport and leading to neuronal death.

As the disease progresses, widespread neuronal loss and brain atrophy become evident, especially in the hippocampus and cortical areas [4]. This neuronal loss is accompanied by a significant shrinkage of the brain, which can be observed as a reduction in brain volume and enlargement of the ventricles on neuroimaging. Even before significant neuronal death, synaptic dysfunction occurs, characterized by reduced synaptic density and impaired neurotransmission, which are major contributors to cognitive decline [9]. Chronic neuroinflammation also plays a key role, with activated microglia and astrocytes surrounding amyloid plaques and neurofibrillary tangles [10]. While these immune cells initially attempt to clear Aβ and damaged neurons, their prolonged activation exacerbates neuronal damage and promotes further disease progression. Additionally, Alzheimer’s disease involves vascular changes, such as cerebral amyloid angiopathy, where Aβ deposits in the walls of cerebral blood vessels weaken them, increasing the risk of microbleeds and reducing the brain’s ability to clear toxins. These structural changes collectively lead to the hallmark symptoms of Alzheimer’s disease, including memory loss, confusion, language difficulties, and impaired reasoning, driving the relentless progression of the disease [4].

In Alzheimer’s disease, brain atrophy follows a characteristic pattern [1,11]. Initially, atrophy begins in the hippocampus and other structures within the medial temporal lobe (i.e., the entorhinal cortex or part of the parahippocampal gyrus). These regions are crucial for memory formation, which correlates with the early symptoms of Alzheimer’s, such as memory loss. As the disease progresses into mild to moderate stages, the atrophy spreads to the inferolateral temporal lobes, indicating that more extensive cortical regions, especially those involved in complex cognitive functions, start to deteriorate [12]. With the further advancement of the disease into moderate to severe stages, atrophy continues to affect the medial parietal lobes, areas associated with visuospatial processing and linked to the disorientation observed in more advanced Alzheimer’s patients. Finally, in the advanced stages, the frontal lobes are impacted [11]. This region governs executive functions such as decision making, personality, and behavior, leading to the severe cognitive and behavioral symptoms characteristic of late-stage Alzheimer’s disease. 

Structural gray matter correlates of Alzheimer’s disease have been identified, with atrophy in key regions, particularly the medial temporal lobe, being a primary marker [11]. This atrophy includes changes in both the volume and cortical thickness, leading to the identification of a specific cortical signature of Alzheimer’s disease [13]. This signature refers to a distinct pattern of cortical thinning that can serve as a biomarker for the disease, distinguishing between cognitively unimpaired individuals and those who either have or are at risk of developing Alzheimer’s disease [13]. Numerous studies have highlighted the critical characteristics of these regions, demonstrating their significant biological implications for the development of cognitive impairment and Alzheimer’s disease [14,15]. Understanding these structural changes is crucial for advancing early diagnosis and potentially intervening before significant cognitive decline occurs.

This review aims to explore the interactions between the mechanisms implicated in the pathogenesis of Alzheimer’s disease and their associations with various microbiome compositions, with a particular focus on the role of physical exercise. We will examine how different microbiome changes are related to the mechanism of Alzheimer’s disease through these critical brain regions and hypothesize how physical exercise could modulate these interactions. Understanding how physical activity impacts the microbiome–brain axis is central to this review and could provide valuable insights into new therapeutic strategies for addressing the complex brain changes associated with Alzheimer’s disease.

## 2. Physical Exercise on Structural Correlates of Alzheimer’s Disease

Physical exercise promotes cognition and improves the symptoms of mood disorders and psychological disorders. Aerobic exercise training has been found to improve cognitive function, as well as the symptoms of depression and schizophrenia [16,17,18]. It has been demonstrated that regular aerobic exercise prevents age-related global brain atrophy and increases the volume of the left superior temporal lobe and frontal lobes, which are essential for cognition, attention, and memory [19]. Similarly, aerobic exercise has been shown to improve (in older adults aged 60–79 years) functional activation in the brain, which enhances efficiency when performing tasks, as well as regulating behavior and mood [20].

In animal models of Alzheimer’s disease, such as APP/PS1 transgenic mice, exercise interventions have been shown to impact the brain regions associated with the disease. For instance, 3 months of voluntary running led to increased volumes in the hippocampal subfields, specifically the dentate gyrus and CA1, compared to sedentary APP/PS1 mice [21]. Similarly, a 12-week treadmill exercise regimen enhanced synapse formation and increased the length and thickness of postsynaptic densities in the hippocampal CA1 region [22]. Furthermore, 10 weeks of treadmill training resulted in increased dendritic arborization in the CA1, CA3, and amygdalar basolateral neurons [23]. However, another study found no effect on medial prefrontal cortex volume after 4 months of running exercise. Despite this, the running exercise prevented the loss of dendritic spines and neurons [24]. These findings support the assumption that exercise may protect against degeneration in Alzheimer’s disease-related brain regions in this animal model.

In human studies, the effects of exercise on Alzheimer’s disease-related brain structures have been studied in older adults, but the findings remain inconclusive, and randomized controlled trials are limited. In healthy older adults, aerobic and coordination training have been associated with increased hippocampal volume [25,26], while resistance training showed no effect [27]. Aerobic exercise has been linked to increased gray and white matter volumes [28], insula cortical thickness [29], and putamen volume [30]. However, resistance training did not yield similar results for cortical gray matter and cortical white matter [27]. In older adults with mild cognitive impairment or at risk of dementia or Alzheimer’s disease, aerobic and multicomponent exercises have been found to increase hippocampal volume [31,32], while a 2-year home-based physical activity program showed no effect [33]. Additionally, high-intensity progressive resistance training protected against hippocampal atrophy one year after a 6-month intervention (three sets of eight repetitions, at 80% of the peak capacity) [34]. While evidence suggests exercise benefits in these populations, the findings are still insufficient for strong conclusions.

Few randomized controlled trials have examined the effects of exercise on brain structures in older adults with Alzheimer’s disease. Frederiksen et al. (2018) evaluated a 16-week aerobic exercise intervention (60 min, three times per week, moderate-to-high intensity) in older adults with mild to moderate Alzheimer’s disease. They found no effects on total hippocampal and subfield volumes, parahippocampal volume, basal ganglia (caudate and putamen) volumes, or regional cortical thickness [35]. However, a positive association was observed between exercise load and changes in hippocampal volume and frontal cortical thickness [35]. Similarly, Morris et al. (2017) assessed a 26-week vigorous aerobic exercise intervention (150 min, three to five times per week, 40–55% to 60–75% of heart rate reserve) in Alzheimer’s disease participants, focusing on hippocampal and gray matter volumes. While no direct effects were found, increased cardiorespiratory fitness was positively associated with bilateral hippocampal volume changes, particularly in the exercise group [36].

These findings indicate limited evidence to draw definitive conclusions about the effects of exercise on brain structures in older adults with Alzheimer’s disease. However, the positive associations between exercise load, cardiorespiratory fitness, and hippocampal volume suggest that further research is needed in this population to clarify these relationships. Although exercise has shown favorable effects on Alzheimer’s disease-related brain structures in healthy older adults and those at risk, research on its impact in patients with Alzheimer’s disease remains limited. Recent meta-analytical evidence indicates that regular exercise or physical activity improves hippocampal volume in humans, preventing volumetric declines over time [37].

Future studies should explore exercise’s effects on brain structure in Alzheimer’s disease patients, recognizing that many older adults with cognitive impairment and Alzheimer’s are capable of and interested in physical activity, which may benefit their brain health [38].

## 3. Gut–Brain Axis in Alzheimer’s Disease

A complex and intricate ecological network, the human microbiome comprises a variety of microorganisms (bacteria, viruses, fungi, and protozoa) that have colonized numerous anatomical locations within the human body, including the skin, oral cavity, vagina, and gastrointestinal tract [39,40]. The microbiome inhabits the human body and interacts with it. Depending on the nature of the interaction, it may be mutualistic, commensalistic, or pathogenic [41,42,43,44,45,46,47]. 

Selective serotonin uptake inhibitors improve gastrointestinal function in individuals with neural disorders [48]. Observations such as these have sparked curiosity among researchers, leading to an increase in research into the connection between the gastrointestinal tract and the brain [49,50,51,52,53].

The gut and the brain are intimately linked during fetal development when the central nervous system and the enteric nervous system are derived from the same tissues [49]. Gut–brain communication is bidirectional and mediated by the autonomic nervous system; efferent and afferent signals through the vagus nerve; neuroendocrine signaling through the hypothalamic–pituitary–adrenal axis; and serotonin regulation [54,55,56,57]. Specific microbiomes can affect other communication channels in the gut–brain axis, such as gamma-aminobutyric acid-glutamate and catecholamines, metabolites of microbial origin, and even hormones [58,59].

**Figure 1:**
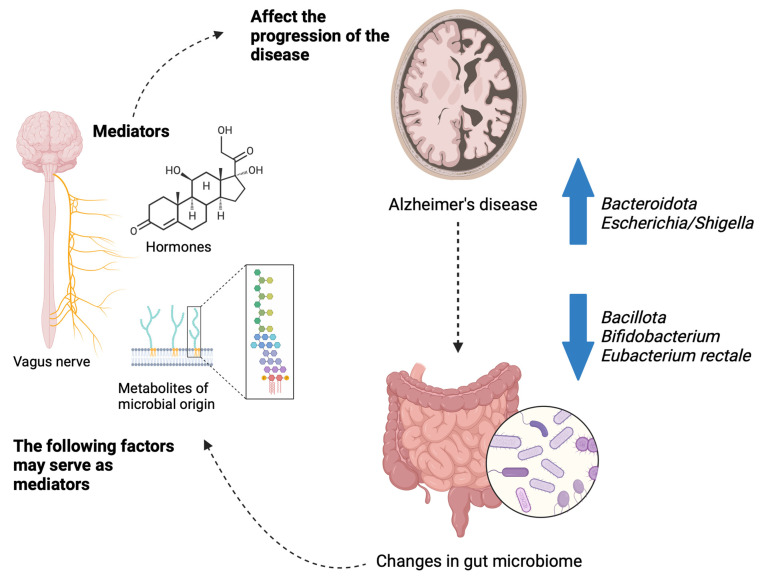
Alzheimer’s disease and the gut microbiome.

Several studies have demonstrated that Alzheimer’s disease participants have different microbiome compositions than cognitively healthy individuals [60,61,62]. There was a decrease in *Bacillota*, an increase in *Bacteroidota*, and a decrease in *Bifidobacterium* abundance in the microbiome of patients with Alzheimer’s disease. Positive correlations were also observed between differentially abundant genera and the biomarkers of Alzheimer’s disease in cerebrospinal fluid according to Vogt et al. [60]. According to another study, bacterial taxa in 43 patients with Alzheimer’s disease differed at taxonomic levels from those in controls, including *Actinobacteriota*, *Bacteroides*, *Ruminococcus*, *Selenomonadales*, and *Lachnospiraceae* [62]. In another study, as a baseline comparison, subjects with normal versus impaired cognition do not exhibit a significant difference in microbiome diversity. However, subjects with mild cognitive impairment display a number of unique microbial signatures. There was a positive correlation between *Pseudomonadota* and Aβ-42: Aβ-40 ratio and a negative correlation between fecal propionate and butyrate and Aβ-42 in subjects with mild cognitive impairment [61]. As compared to both healthy controls and patients without brain amyloidosis, patients with cognitive impairment had lower abundances of *Eubacterium rectale* and higher abundances of *Escherichia*/*Shigella*. The abundance of *Escherichia*/*Shigella*, an inflammatory bacteria taxon, was positively correlated with the presence of pro-inflammatory cytokines, such as interleukin (IL)-1β, whereas *Eubacterium rectale*, an anti-inflammatory bacteria taxon, was negatively correlated with the presence of those pro-inflammatory cytokines [63]. A diagram of the gut microbiome in Alzheimer’s disease can be seen in Figure 1.

## 4. Gut–Brain Axis and Exercise in Alzheimer’s Disease

Studies have shown that physical exercise and gut–brain axis play a significant role in the treatment and prevention of Alzheimer’s disease in both humans and animals. Below is a summary of these studies.

### 4.1. Animal Studies of Alzheimer’s Disease, Exercise, and the Gut–Brain Axis 

In rats with a disrupted microbiome, performance on two hippocampal neurogenesis-dependent tasks was impaired. These tasks were the novelty-suppressed feeding test and the modified spontaneous location recognition task. Physical exercise mitigated the reduction in hippocampal neurogenesis caused by gut microbes in these rats [64]. A treadmill running protocol composed of 10 cycles of 6 min at high intensity, followed by two minutes at low intensity for two weeks resulted in increased antioxidant defenses and improved anti-inflammatory system performance in Alzheimer’s disease transgenic mice. As a result of these effects, pro-apoptotic proteins in intestinal lymphocytes appear to be reduced, and short-chain fatty acid (SCFA)-producing bacteria and *Lactobacillus reuteri*, a vitamin B_12_ producer, are increased, which improves cognitive function and slows the progression of biomarkers such as Aβ-42, total tau, and phosphorylated tau [65].

As a result of forced treadmill running, the gut microbiome changes in a symbiotic manner. This is due to an increase in *Akkermansia muciniphila* and a decrease in *Bacteroides* species. In addition, there is an increase in the blood–brain barrier-related protein expression and a reduction in the progression of Alzheimer’s disease-like cognitive impairments [66].

Through the production of palmitoleic acid, which is protective against inflammation and metabolic disorders, *Dubosiella* enrichment hinders the progression of Alzheimer’s disease in an animal model. The correlation between deoxycholate levels and cognitive scores in humans has also been supported by fecal deoxycholic acid-mediated interactions between the Alzheimer’s disease hub bacteria *Erysipelatoclostridium* and the disease’s occurrence [67]. In male APP/PS1 transgenic mice, exercise-induced changes in the microbiome do not significantly affect mitochondrial density and the AMPK/mTOR/S6 pathways related to protein synthesis [68]. Physical exercise has a positive effect on other organs, making Alzheimer’s disease a systemic disease [69,70].

Exercise reduced disintegrin and metalloproteinase domain-containing protein 10 and glial fibrillary acidic protein expression in the hippocampus of middle-aged APP/PS1 mice, but there were no significant alterations in circulating metabolites. Moreover, mice from the exercise group had markedly reduced abundances of the phyla *Pseudomonadota* and *Tenericutes*, genera *Bacteroides* and *Faecalibacterium*, and elevated abundances of the genus *Allobaculum* [71]. Exercise increased the alpha diversity index of cecal content in APP/PS1 mice, and trimethylamine N-oxide and exercise had a differential effect on gut microbiome profiles [72].

### 4.2. Human Studies of Alzheimer’s Disease, Exercise, and the Gut–Brain Axis

There is a link between Alzheimer’s disease and changes in the microbiome, and exercise appears to be beneficial in treating the disease. Moreover, physical activity is beneficial to the human intestinal microbiome. Therefore, this complex interaction may be advantageous for patients with Alzheimer’s disease as well as for the professional community and national budgets [73]. Next, we will present the results of a multimodal lifestyle intervention in humans that included exercise.

A 1:1 multicenter, phase 2 randomized controlled study on multimodal lifestyle intervention (including exercise, diet, and stress management, among others) was conducted on fifty-one patients with mild cognitive impairment or early dementia due to Alzheimer’s disease. The exercise component of the intervention included aerobic exercises (e.g., walking) for at least 30 min per day and strength training three times per week, either in person or via virtual consultations with an exercise physiologist. Based on the patient’s age and fitness level, an exercise prescription was developed for 20 weeks [74]. *Blautia*, which was found to increase in the intervention group during the intervention, has previously been associated with a lower risk of Alzheimer’s disease, possibly because it promotes gamma-aminobutyric acid production [75]. During the intervention, *Eubacterium* also increased in the intervention group, and prior studies have demonstrated that the *Eubacterium* genus (specifically *Eubacterium fissicatena*) protects against Alzheimer’s disease [76]. Furthermore, the relative abundance of the taxa associated with an increased risk of Alzheimer’s disease decreased in the intervention group, e.g., *Prevotella* and *Turicibacter*, the latter of which has been associated with relevant biological processes such as serotonin production [74]. Previously, *Prevotella* and *Turicibacter* rates increased with disease progression [77], but these rates decreased after the intervention. Despite these insights, research on the intricate effects of physical exercise on the gut–brain connection in humans remains relatively scarce. There is a need for human studies that evaluate only exercise as the main variable. The existing studies have primarily focused on isolated aspects rather than the comprehensive interactions between exercise, gut health, and cognitive function. The main studies discussed in this section are summarized in Table 1.

In the following section, we will present a detailed theoretical framework that can serve as a foundation for future research. This framework aims to integrate the existing knowledge and guide new studies in examining how physical exercise influences the gut–brain axis, with the goal of uncovering more nuanced and actionable insights.

## 5. Mechanisms Involved

Exercise’s effect on the gut microbiome is believed to contribute to the treatment or prevention of Alzheimer’s disease in several ways, including modulating neuroinflammation, amyloid metabolism, brain-derived neurotrophic factor release, and the transport of gut-derived metabolites across the blood–brain barrier. 

### 5.1. Inflammation Reduction—Chronic Inflammation

A gut–brain axis pathway may be responsible for communication between the gut microbiome and the nervous system, which includes vagus nerve neuronal connections, the enteric nervous system, metabolism, and the immune system [78]. Exercise could modulate the gut microbiome through mechanisms that are closely related to an anti-inflammatory role, which results in a reduced level of inflammation in the gut [79]. Several mechanisms underlie exercise-induced benefits for cognition, including the gut microbiome regulation of neurogenesis and neuroinflammation [80,81]. Exercise has a neuroprotective effect, enhancing angiogenesis and neurogenesis, reducing inflammation, and reducing cerebrovascular risk factors [82,83,84]. Exercise programs that are conducted over a long period of time can reduce the risk factors for Alzheimer’s disease, improve blood flow, increase hippocampal volume, and improve neurogenesis in the brain, including swimming, walking, cycling, yoga, and bowling [85].

### 5.2. Amyloid Metabolism

There is evidence to support the belief that decreased Aβ clearance is an integral part of the pathomechanism of Alzheimer’s disease [86]. In the early stages of amyloid plaque accumulation, memory problems are the most common clinical symptom [85]. Additionally, Alzheimer’s disease may affect metabolism through the accumulation of pathological amyloid plaques in peripheral organs [87,88,89,90]. Based on these observations, Alzheimer’s disease is considered to be a complex systemic disease [70,91].

The gut microbiome contains several species of bacteria that can produce amyloid peptides, including *Escherichia coli*, *Citrobacter*, *Mycobacteria*, *Klebsiella*, *Pseudomonas*, *Staphylococcus*, *Streptococcus*, *Streptomyces*, *Salmonella*, and *Bacillus* [92,93]. There is a possibility that these peptides can be transmitted to the brain and accumulate there, leading to cognitive dysfunction [94]. The biomarkers of Alzheimer’s disease are also associated with a reduction in *Bifidobacterium* and *Eubacterium rectale* [95]. Despite the absence of any clear causal relationship between the gut microbiome and neurodegeneration, these results suggest that it may play an important role in the pathogenesis of dementia [96]. According to a study that compared the gut microbiome of amyloid-positive patients, amyloid-negative patients, and healthy controls, amyloid-positive patients had a low ratio of *Eubacterium rectale* and a high proportion of *Escherichia*/*Shigella* [97]. By regulating the immune system, differences in the gut microbiome influence amyloid accumulation in the brain [63]. While animal studies show that amyloid clearance is one of the possible mechanisms by which exercise positively impacts brain health [98,99,100], only a few human studies have been conducted and the results show no effect [101] or remain inconclusive [102].

### 5.3. Brain-Derived Neurotrophic Factor

Multiple studies have demonstrated that brain-derived neurotrophic factor is required for neurogenesis in the subventricular zone of the hippocampus [103,104,105]. Additionally, to modulate neurogenesis, brain-derived neurotrophic factor plays an important role in brain physiology, as well as in muscle tissue and vascular endothelial cells outside of neuronal tissue [103,104,105]. This neurotrophic factor play an important role in long-term potentiation and synaptic plasticity in the central nervous system, where it influences the morphology of mature neurons by promoting the growth of axons, the formation of dendritic arbors, and the pruning of dendrites [103,104,105,106].

Brain-derived neurotrophic factor expression was strongly negatively correlated with germ-free mice and at the phylum level of *Bacillota*, *Bacteroidota*, and *Pseudomonadota*. *Bacillota* also showed a negative correlation with neurogenesis. It is clear that the loss of healthy gut microbiome, such as in germ-free mice, has a detrimental effect on the brain, but it remains unclear why both the major phyla of the healthy microbiome (*Bacillota* and *Bacteroidota*) showed strong negative correlations [107]. Therefore, it is important to take precautions when interpreting the results at the phylum level. In addition, multiple topics concerning the role of the microbiome in neurogenesis and brain-derived neurotrophic factor expression were identified, including age, obesity, chronic stress, and antibiotic treatment. There has, however, been difficulty in making a definitive connection between a bacterial taxon and brain-derived neurotrophic factor expression or neurogenesis in adult animals, which sometimes leads to results that are inconclusive. For this purpose, studies with gnotobiotic mice that focus their attention on one taxonomic rank of bacteria would be helpful in determining the individual interactions between the microbiome, the gut, and the brain [106].

The presence of high levels of a brain-derived neurotrophic factor in the brain has been linked to improvements in memory and recollection, as well as the prevention of cognitive decline [108,109] and the brain-derived neurotrophic factor is one of the main candidates by which exercise exerts a positive effect on the brain [110,111]. In contrast, decreased levels of brain-derived neurotrophic factor are associated with poor memory function, neurodegeneration, and cognitive impairments associated with Alzheimer’s disease [112].

### 5.4. Transport of Gut-Derived Metabolites across the Blood–Brain Barrier

Previous studies have shown that the gut is crucial for the release of hormones, peptides, and microbial metabolites, including SCFAs, secondary bile acids, and products derived from tryptophan and polyphenols. Neuronal function and survival are significantly affected by these substances. Several of these compounds can cross the blood–brain barrier, including SCFAs, which use active membrane transporters in the endothelium to reach the central nervous system [113]. There are enteroendocrine cells within the enteric nervous system which are responsible for receiving signals from the gut microbiome directly. By secreting hormones, these cells can influence the function of brain cells across the blood–brain barrier [114].

Enteroendocrine cells are intricately connected with the vagus nerve, serving as a possible link between the gut microbiome and the brain [115]. It is possible to communicate bidirectionally between the gut and the brain via this direct connection, allowing for the exchange of signals and information that can influence a variety of physiological and neurological processes [116]. Due to the vagus nerve’s role in this communication pathway, it is important that exercise facilitates interactions between the gut microbiome and the brain, which may result in the vagus nerve mediating the gut–brain axis [117].

A number of immuno-signaling mediators, including cytokines, chemokines, and microbial-associated molecular patterns, contribute to the facilitation of communication between the gut microbiome and the brain [114]. This includes microbial constituents such as lipopolysaccharides and peptidoglycans [118]; microbial products such as enzymes, SCFAs, and neurotransmitters [119,120]; hormone release (glucocorticoids) [121]; and substrate metabolism (bile acids and tryptophan) [122,123]. 

Direct and indirect pathways are available for interaction between these mediators, allowing bidirectional signaling between the brain and the gut [124]. Using these signaling pathways, the gut microbiome can influence immune responses and inflammation in the brain, as well as modulate immune function in the gut through the brain [114,125]. 

**Figure 2:**
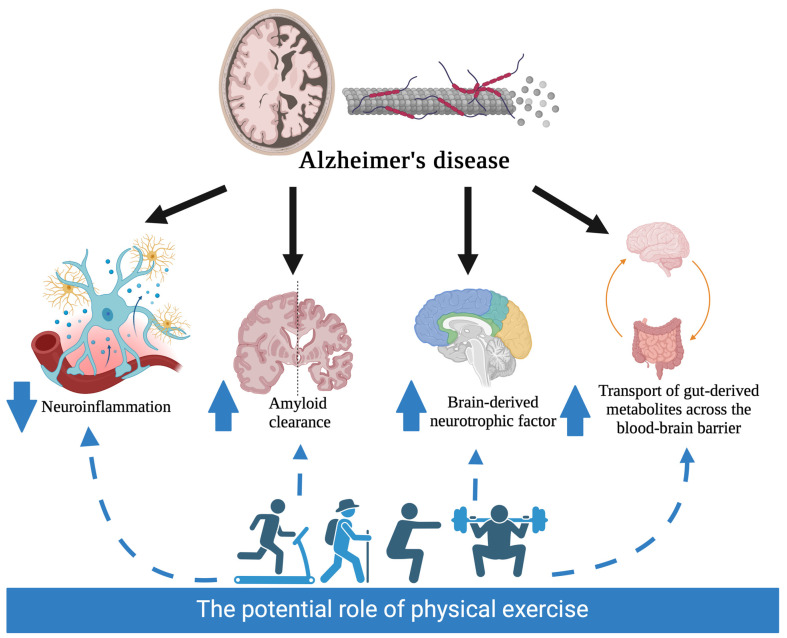
Involvement of physical activity in Alzheimer’s disease mechanisms. A black arrow indicates the mechanism implicated in Alzheimer’s disease, and a blue arrow indicates the impact of physical activity on the disease.

Interestingly, the potential for physical exercise to modify blood–brain barrier permeability has been previously discussed [126], supported by a clear theoretical rationale that positions it as a significant factor in this phenomenon. This connection suggests that exercise may play a crucial role in maintaining the integrity of the blood–brain barrier, which is vital for neurological health. Figure 2 illustrates the mechanism described in the manuscript.

## 6. Future Directions

A multitude of mechanisms associated with Alzheimer’s disease can be modified by physical exercise. The gut–brain axis as a potential pathway to understand the effect of exercise on brain structure and the final behavioral consequences is promising [85]. The performance of cognitive functions, memories, and executive functions can be improved through a variety of activities, including swimming, walking, cycling, yoga, and bowling. Additionally, an hour-long resistance exercise program can help reduce the progression of Alzheimer’s by improving physical function long term [127,128]. 

Despite this, in some of the mechanisms proposed, the understanding is limited due to a variety of reasons. These include the lack of human studies, the lack of a targeted approach to the understanding of this dual mechanism, or the lack of diversity of interventions (in terms of type, duration, and population, among others). This document sheds light on the targeted approach to the gut–brain axis as a potential key mechanism in the understanding of the complexity of the disease with the final aim of improving the targeted approach to the disease and more effective disease-modifying therapies.

## Figures and Tables

**Table 1:** Research on the gut–brain axis and exercise in Alzheimer’s disease.

Reference	Protocol for Exercise	Alzheimer’s Disease and the Microbiome
Nicolas, S. et al. [64]	For ten weeks, rats were housed in pairs in either standard housing cages or cages that provided continuous and free access to running wheels.	These rats were able to mitigate the reduction in hippocampal neurogenesis caused by gut microbes through physical exercise.
Abraham, D. et al. [65]	Ten cycles of 6 min at high intensity, followed by two minutes at low intensity for two weeks in mice.	There was an increase in *Lactobacillus reuteri* and bacteria that produce short-chain fatty acids, which resulted in improved cognitive function and slowed the progression of Alzheimer’s disease biomarkers.
Jin, K. et al. [66]	A motor-driven treadmill for rodents was used to train mice five days a week for 18 weeks. In each session, a warm-up period of five minutes at a speed of five meters per minute was followed by five minutes at a speed of eight meters per minute and a maximum speed of eleven meters per minute for twenty minutes. Once the maximum speed reached 15 m/min, it was increased by 1 m/min every four weeks.	There has been an increase in *Akkermansia muciniphila* and a decrease in *Bacteroides* species. A reduction in the progression of Alzheimer’s disease-like cognitive impairment is also observed, as well as an increase in the blood–brain barrier-related protein expression.
Teglas, T. et al. [68]	The training was conducted four times a week for 60 min in mice. There were ten cycles of training, each consisting of four minutes at a high intensity (20 m/min) and two minutes at a low intensity (10 m/min).	Changes in the microbiome caused by exercise do not significantly affect the mitochondrial density and the AMPK/mTOR/S6 pathways related to protein synthesis.
Wang, G. et al. [71]	The running wheel was accessed freely and unlimitedly by mice	*Pseudomonadota* and *Tenericutes*, *Bacteroides*, and *Faecalibacterium* were markedly reduced in the exercise group, while *Allobaculum* was increased
Zhang, Y. et al. [72]	The running wheel was accessed freely and unlimitedly by mice	In APP/PS1 mice, exercise increased the alpha diversity index of cecal content
Ornish, D. et al. [74]	A 20-week program of aerobic exercise (e.g., walking) and strength training exercises in humans	There was an increase in *Blautia* and *Eubacterium* in the intervention group during the intervention. Additionally, in the intervention group, the relative abundance of the taxa associated with an increased risk of Alzheimer’s disease decreased, e.g., *Prevotella* and *Turicibacter*
